# Supplementary material for: Effects of Organic Elicitors on the Recycled Production of Ginkgolide B in Immobilized Cell Cultures of Ginkgo biloba
Source: J Funct Biomater. 2023 Feb 9;14(2):95. doi: 10.3390/jfb14020095 (PMC9966486; doi:10.3390/jfb14020095)
Supplement: Supplementary file 1 [file jfb-14-00095-s001.zip › jfb-2182591-Supplementary.pdf]

Supplementary Materials

S1

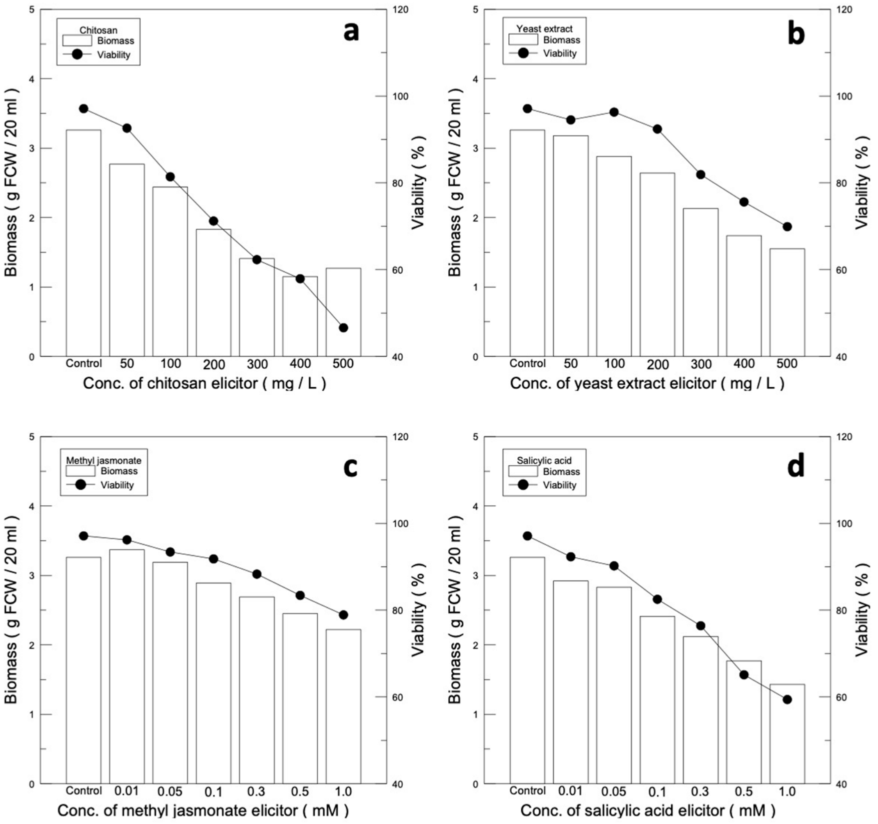

Figure S1. Comparison of cell growth and cell viability of ginkgo suspension cells with different concentrations of 4 individual elicitors. (a) Chitosan; (b) Yeast extract; (c) Methyl jasmonate; (d) Salicylic acid.

S2

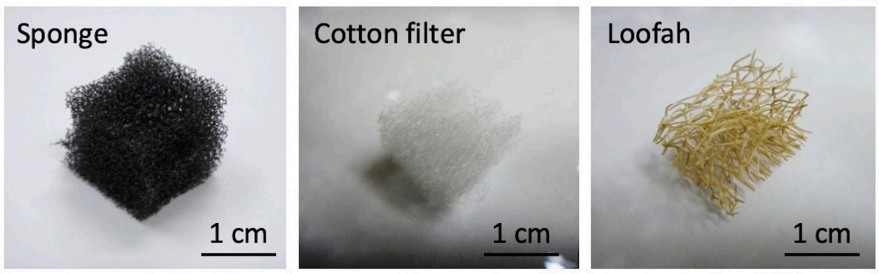

Figure S2. the images of immobilized materials.

S3

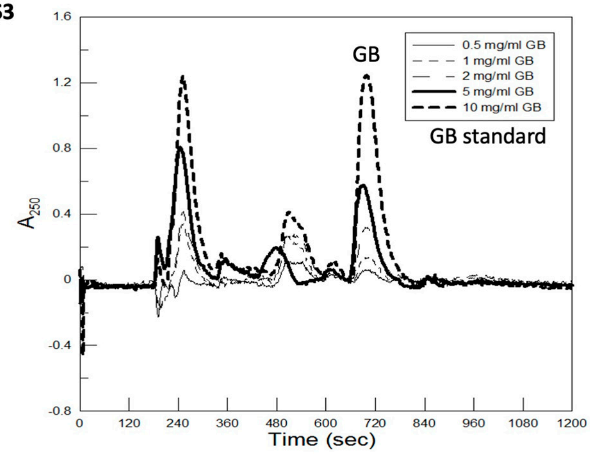

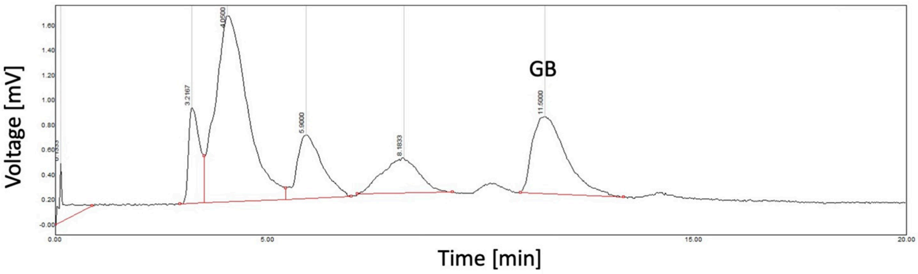

Figure S3. HPLC chromatograms of the GB standard and the extract.
